# Supplementary material for: Age of Cafeteria Diet Onset Influences Obesity Phenotype in Mice in a Sex-Specific Manner
Source: Int J Mol Sci. 2024 Nov 19;25(22):12436. doi: 10.3390/ijms252212436 (PMC11595127; doi:10.3390/ijms252212436)
Supplement: Supplementary file 1 [file ijms-25-12436-s001.zip › Table S1.pdf]

Table S1. Parameters measured in male and female mice of control, 7CafD and 17CafD groups at 27 weeks of age.

**I. Lipid metabolism**				
1. Weight parameters	Body weight, relative WAT and liver weight			
2. Blood parameters	leptin, adiponectin, triglycerides, FFA, cholesterol			
3. Hepatic parameters	triglycerides			
4. Gene expression	Liver	Muscle	WAT	BAT
▪ Fatty acid oxidation	*Pparα, Ppargc1, Cpt1α*	*Cpt1β, Ucp3*	*Pparγ, Ppargc1, Cpt1α*	
▪ Lipolysis			*Lipe, Pnpla2*	
▪ Lipogenesis	*Fasn, Acca, Accb*		*Fasn, Lpl*	
▪ Lipoprotein synthesis	*Apob*			
**II. Carbohydrate metabolism**				
1. Blood parameters	glucose, insulin			
2. Hepatic parameters	glycogen			
3. Gene expression	Liver	Muscle	WAT	BAT
▪ Insulin signaling	*Insr, Irs1, Irs2*	*Insr, Irs1, Irs2, Slc2a4*	*Insr, Slc2a4*	
▪ Glycolisis	*Gck, Pklr*			
▪ Gluconeogenesis	*Pck1, G6pc*			
**III. Thermogenesis**				
1. Weight parameters	relative BAT			
2. Gene expression	Liver	Muscle	WAT	BAT
▪ Thermogenesis			*Ucp1, Dio2*	*Ucp1, Dio2*
▪ Fatty acid oxidation				*Pparγ, Cpt1β*
***IV.* FGF21 system activity**				
1. Blood parameters	FGF21			
2. Gene expression	Liver	Muscle	WAT	BAT
▪ FGF21 system activity	*Fgf21, Klb*	*Fgf21, Klb*	*Fgf21, Klb*	*Fgf21, Klb*
