# Supplementary figures and images for: Age of Cafeteria Diet Onset Influences Obesity Phenotype in Mice in a Sex-Specific Manner
Source: Int J Mol Sci. 2024 Nov 19;25(22):12436. doi: 10.3390/ijms252212436 (PMC11595127; doi:10.3390/ijms252212436)

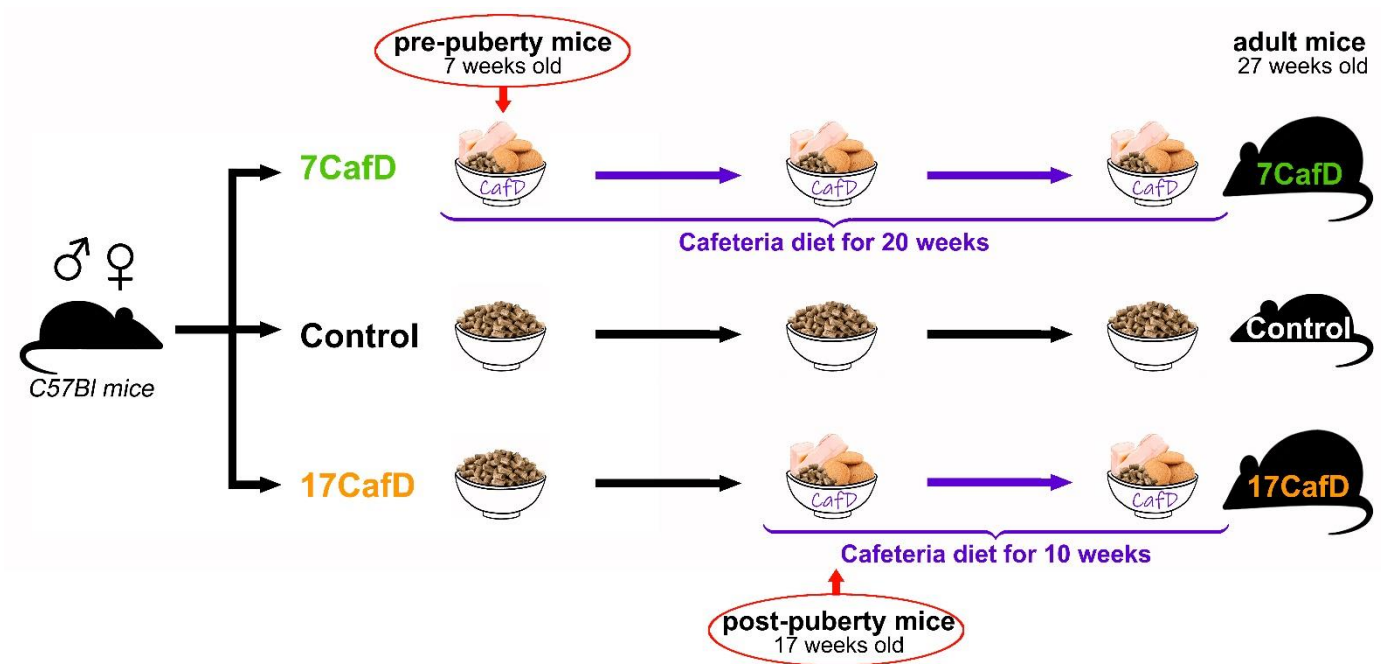

**Figure S1.** Experimental design.

Supplement: Supplementary file 1 [file ijms-25-12436-s001.zip › Figure S1.pdf]
